# *Plasmodium vivax*: comparison of immunogenicity among proteins expressed in the cell-free systems of *Escherichia coli *and wheat germ by suspension array assays

**DOI:** 10.1186/1475-2875-10-192

**Published:** 2011-07-14

**Authors:** Edmilson Rui, Carmen Fernandez-Becerra, Satoru Takeo, Sergi Sanz, Marcus VG Lacerda, Takafumi Tsuboi, Hernando A del Portillo

**Affiliations:** 1 Barcelona Centre for International Health Research (CRESIB), Hospital Clinic/IDIBAPS, Universitat de Barcelona, Roselló 153, 1a planta, 08036, Barcelona, Spain; 2 Cell-Free Science and Technology Research Center, Ehime University, Matsuyama, Ehime 790-8577, Japan; 3 Fundação de Medicina Tropical Dr. Heitor Vieira Dourado, Manaus, Brazil; 4 Venture Business Laboratory, Ehime University, Matsuyama, Ehime 790-8577, Japan; 5 Institució Catalana de Recerca I Estudis Avançats (ICREA), Barcelona, Spain

## Abstract

**Background:**

In vitro cell-free systems for protein expression with extracts from prokaryotic (*Escherichia coli*) or eukaryotic (wheat germ) cells coupled to solid matrices have offered a valid approach for antigen discovery in malaria research. However, no comparative analysis of both systems is presently available nor the usage of suspension array technologies, which offer nearly solution phase kinetics.

**Methods:**

Five *Plasmodium vivax *antigens representing leading vaccine candidates were expressed in the *E. coli *and wheat germ cell-free systems at a 50 μl scale. Products were affinity purified in a single-step and coupled to luminex beads to measure antibody reactivity of human immune sera.

**Results:**

Both systems readily produced detectable proteins; proteins produced in wheat germ, however, were mostly soluble and intact as opposed to proteins produced in *E. coli*, which remained mostly insoluble and highly degraded. Noticeably, wheat germ proteins were recognized in significantly higher numbers by sera of *P. vivax *patients than identical proteins produced in *E. coli*.

**Conclusions:**

The wheat germ cell-free system offers the possibility of expressing soluble *P. vivax *proteins in a small-scale for antigen discovery and immuno-epidemiological studies using suspension array technology.

## Background

The recent call for malaria eradication has re-emphasized the importance of bringing *Plasmodium vivax *into the research agenda [1]. *Plasmodium vivax *remains the most widely distributed human malaria parasite with 2.85 billion people living at risk of infection [2]. Noticeably, the number of yearly clinical cases seems to be increasing from 70-80 million [3] to 300 million cases [4] and these include cases of severe disease and death exclusively associated with *P. vivax *[5,6]. Moreover, experts agree that present tools against *Plasmodium falciparum *will not be effective against *P. vivax*, reinforcing the development of control measurements for this species [7]. Among these tools, vaccines continue to represent the most cost-effective control measurement but unfortunately vaccine development in *P. vivax *lags well behind that of *P. falciparum *[8].

The genomes of human malaria parasites encode approximately 5,400 coding genes opening an avenue for antigen discovery in this species [9]. Unfortunately, cell-based expression systems have met limited success to obtain soluble proteins largely attributed to the high AT-content, the existence of long stretches of repeated amino acid sequences and much larger proteins than their homologues in other eukaryotes [10]. In contrast to cell-based systems, cell-free expression systems for protein synthesis with extracts from prokaryotic or eukaryotic cells has offered a valid alternative to express soluble proteins [11]. In the case of malaria, using the *Escherichia coli *cell-free system, Doolan and co-workers first reported on the expression of 250 *P. falciparum *proteins subsequently coupled to solid arrays and analysed with immune sera discovering putative new antigens [12]. Using this same approach, expression of 1,204 *P. falciparum *proteins later expanded these analysis and predicted new antigens [13]. Parallel efforts were reported on the use of cell-free extracts from wheat germ to similarly produce hundreds of *P. falciparum *proteins [14,15]. More recently, the wheat germ expression system has been used for antigen discovery in *P. vivax *[16]. Thus, 89 different soluble proteins were expressed and shown to be immunogenic on analyses of protein arrays and immune sera. Together, this data demonstrates that cell-free expression systems coupled to protein arrays offer a scalable platform for antigen discovery in malaria.

Suspension array technologies with high-throughput capacity to simultaneously analyse several proteins with minimal amount of immune sera have also been developed and used in analysis of multiple malaria vaccine candidates as well as in developing functional assays [17-20]. Suspension arrays offer several advantages as compared to flat protein arrays including nearly solution phase kinetics and total assay sensitivity [21]. The aim of this study was to develop a small-scale method for soluble expression of *P. vivax *proteins using the *E. coli *and wheat germ cell-free systems and to compare their usage by multiplexing assays.

## Methods

### Human samples

Human plasma samples were obtained from endemic areas of Brazil and from a non-endemic region. The first group comprised immune sera from adults living in the Brazilian Amazon [22]. The other group comprised sera from four healthy adult volunteers living in the city of Barcelona (Spain) that have never been exposed to malaria or visited malaria endemic regions. These studies received the ethical approval of Local Institutional Reviewing Boards.

### Construction of plasmids

**Figure 1:**
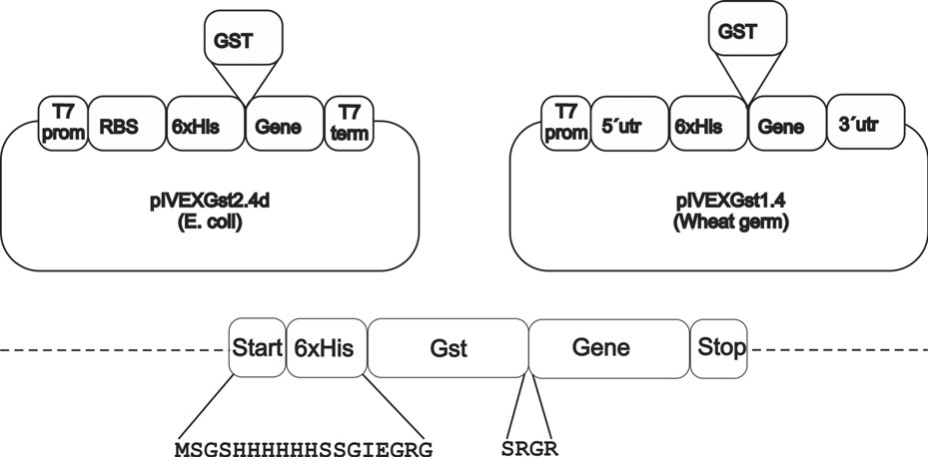
**Construction of recombinant expression vectors**. Expression vectors for wheat germ (pIVEX1.4) and *E. coli *(pIVEX2.4) were originally purchased from Roche. GST was subsequently introduced into these vectors between the 6xHis tag and the multiple cloning site (MCS) to generate plasmids pIVEGST1.4 and pIVEXGST2.4. Amino acids between GST and *P. vivax *proteins are the same for all constructs.

**Figure 2:**
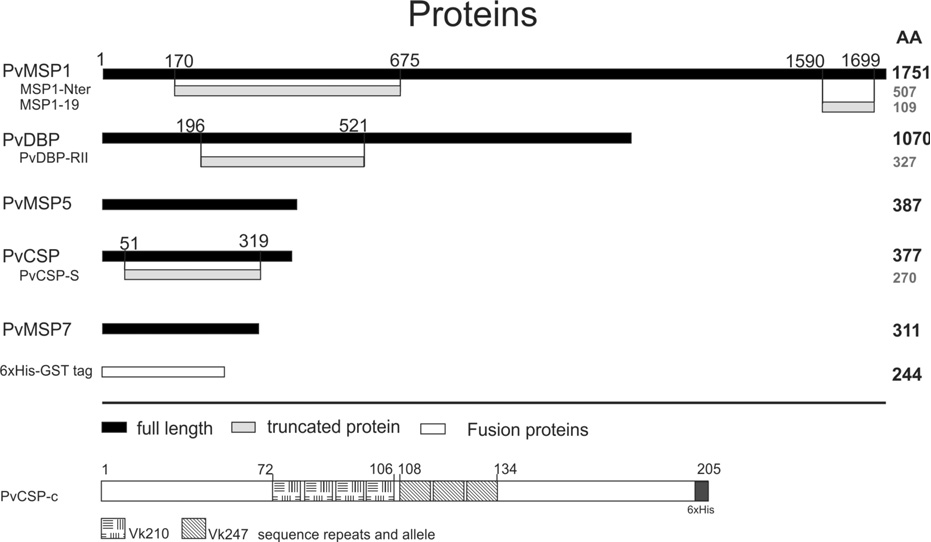
**Schematic representation of recombinant proteins expressed in *E. coli *and wheat germ**. Merozoite surface protein 1 N-terminus (MSP1-Nter), Merozoite surface protein 1 C-terminus (MSP1-19), Duffy binding protein - region II (PvRDBP-RII), Merozoite surface protein 5 (PvMSP5), Circumsporozoite protein - Salvador strain (PvCSP-S), Merozoite surface protein 7 (PvMSP7), 6His-tagged Gluthatione-S-transferase (6His-GST), Circumsporozoite protein - chimeric (PvCSP-c). Numbers indicate amino acid (aa) residues. Predicted sizes of recombinant proteins in aa are shown to the right.

Plasmid pIVEX1.4d for expression in wheat germ and pIVEX2.4d for expression in *E. coli *were purchased from Roche and modified by inserting GST after the 6xHis tag sequence. Modified plasmids were termed pIVEXGST1.4d and pIVEXGST2.4d (Figure 1). Both vectors carry the same T7-DNA promoter elements, the ampicillin selectable marker and identical His-GST tags in the same positions. The following proteins were engineered into these vectors: PvMSP1-19 (1590-1699 aa, id PVX_099980) and PvMSP1-Nter (170-675 aa, id PVX_099980); PvDBP-RII (196-521 aa, id PVX_110810); PvCSP-S (51-319 aa, id PVX_119355); PvMSP5 (full length, id PVX_003770); PvMSP7 (full length, id PVX_082695 (Figure 2). Further information on these proteins and primers used for amplifications can be obtained as supplementary information (Additional file 1).

**Figure 3:**
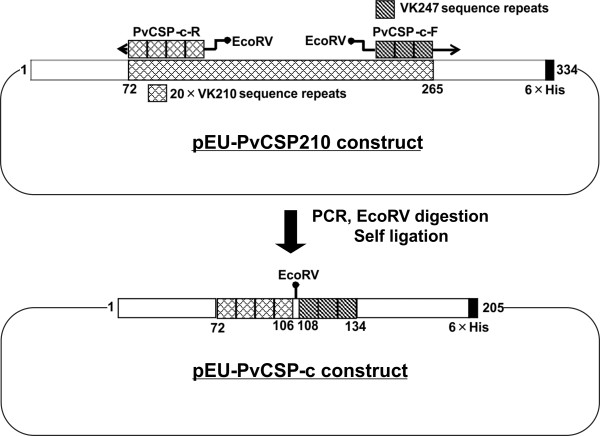
**Schematic representation of the cloning strategy to produce a chimeric circumsporozoite protein contaning canonical major repeats**. Recombinant chimeric PvCSP protein containing VK210-(PVX_119355) and VK247-type (GenBank#M69059, *P. vivax *PNG strain) amino acid repeat sequences (PvCSP-c).

The circumsporozoite antigen of *P. vivax *is dimorphic based on the central repeat region and the two alleles, VK210- and VK247-type, share no immunological cross-reactivity [23]. Therefore, a recombinant chimeric PvCSP protein containing VK210-(PVX_119355) and VK247-type (GenBank#M69059, *P. vivax *PNG strain) amino acid repeat sequences (PvCSP-c) which may cover the vivax parasite population globally was developed (Figure 3). The PvCSP-c was constructed and expressed in a large-scale wheat germ cell-free system (CellFree Sciences, Matsuyama, Japan). Briefly, the nucleotide sequences of PvCSP (SalI strain, VK210 type: PVX_119355) excluding the signal peptide and the GPI anchor signal, with addition of penta-His-tag sequence at the C-terminus, was amplified from SalI gDNA by PCR using VK210-F and VK210-R primers, and was cloned at the EcoRV site into the pEU-E01-MCS plasmid (CellFree Sciences) in the presence of both EcoRV restriction enzyme and T4 DNA ligase generating the pEU-PvCSP210 construct without original EcoRV site. The pEU-PvCSP210 was then inversely amplified by PCR using antisense-primer encoding the four times of the VK210-repeat amino acid sequence "GQPAGDRAD" at the 5' end with EcoRV site (PvCSP-c-R) (Additional file 1) and sense-primer encoding the three times of the VK247-repeat amino acid sequence "GANGAGNQP" at the 5' end with EcoRV site (PvCSP-c-F) (Additional file 1). Then the PCR product was digested with EcoRV, and self ligated after the gel-purification of the restricted DNA fragment. Finally, the presence of tetra-VK210-type sequence was confirmed followed by tri-VK247-type repeat amino acid sequences after the nucleotide sequencing of the final pEU-PvCSP-c plasmid (Figure 3). Deduced amino acid sequences, Gly_2 _to Asp_106 _and Asn_134 _to Cys_199 _in PvCSP-c was identical to Gly_23 _to Asp_127 _and Asn_286 _to Cys_347 _based on the *Sal*I sequence, PVX_ 119355, and Gly_108 _to Pro_134 _in PvCSP-c was identical to Gly_248 _to Pro_274 _based on the deduced amino acid sequence from *P. vivax *PNG strain, M69059.

### In vitro protein synthesis

In vitro protein synthesis followed the original manufacturers' instructions (Roche) and was done on a 50 μl scale, excepting for PvCSP-c (see below). Expressed proteins were purified on GST SpinTrap purification columns (GE Healthcare). Briefly, soluble fractions from cell-free system extracts were applied to a Glutathione Sepharose^® ^4B column that had been equilibrated with PBS. The column was washed with PBS and the bound GST-HBx fusion protein was eluted with 10 mM glutathione in 50 mM Tris-HCl, pH 8.0. Eluted proteins were extensively dialyzed in PBS to remove glutathione. Proteins were analysed by SDS-page and Western blot and quantified as described else where [24]

### Larger scale wheat germ cell-free protein synthesis

The recombinant PvCSP-c protein was synthesized with the wheat germ cell-free protein expression system using the bilayer translation reaction method on a 30 ml scale as manufacturer's recommendation (CellFree Sciences) [14]. The PvCSP-c protein was affinity purified by Ni-affinity chromatography as described previously [25]. Briefly, add imidazole (pH 8.0) in the translation reaction mixture (final concentration, 20 mM) and then add Ni-NTA beads (QIAGEN, Valencia, CA). Incubate the tube for 16 h on a continuous rotator, at 4°C, for the binding of proteins on to the beads. Transfer the solution with the beads into a Poly-Prep chromatography column (Bio-Rad, Hercules, CA). Wash the beads by five bed-volumes of PBS containing 30 mM imidazole three times and then elute the recombinant protein with one bed-volume of PBS containing 500 mM imidazole.

### Covalent coupling of recombinant proteins to beads

BioPlex carboxylated beads (Bio-Rad) were covalently coated with the different recombinant proteins following the manufacturer's instructions (BioPlex Amine Coupling Kit). Briefly, activated beads (1.25 × 10^6 ^beads) were resuspended in 100 μl of PBS and 1 μg of each recombinant protein used per coupling reaction. Incubation under rotation was done at 4°C overnight and coupled beads were washed with 500 μl of PBS pH 7.4. After re-suspending coupling beads in 250 μl of blocking buffer and further incubation under rotation at room temperature for 30 min, beads were washed with 500 μl of storage buffer and centrifuged for six minutes at 14,000 × g. Pellets were resuspended into 125 μl of the same buffer and stored at 4°C protected from light until use.

### Analysis of coupled beads on the BioPlex system

Coupled beads were analysed in the Bioplex system as previously described [20] with slight modifications. Briefly, circa 3,000 coated beads were used for each assay. Frozen plasma samples were thawed at room temperature, diluted 1:50 in assay buffer and 50 μl aliquots added to the beads (final plasma dilution 1:100). Aliquots of 50 μl of Biotinylated human IgG antibody (Sigma) diluted 1:10,000 and of phycoerythrin conjugated streptavidin diluted to 1 μg/ml were used in subsequent incubations. Beads were re-suspended in 125 μl of assay buffer (BioRad) and analysed on the BioPlex100 system and results were expressed as median fluorescent intensity (MFI).

### Statistical analysis

T-test and chi-square or fisher exact test were used to compare mean levels for prevalence, respectively, between groups. Averages were expressed as geometric mean (GM) plus 95% confidence intervals (CI). To evaluate the statistical measure of agreement between two independent proteins the index Kappa was calculated.

## Results

### Cloning and expression of *Plasmodium vivax *proteins

**Figure 4:**
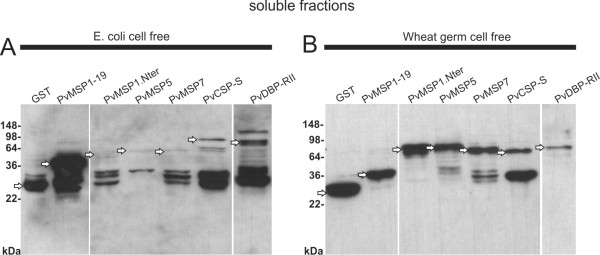
**Expression of *Plasmodium vivax *proteins in cell-free systems**. A. Expression of proteins in the *E. coli *cell-free system. B. Expression of proteins in the wheat germ cell-free system. Both extracts were separated in soluble and insoluble protein fractions by centrifugation (14,000 g/20 mim/4°C). Soluble fractions were analysed by Western blot using anti-GST (HRP) antibody. Molecular weights in kilo-Daltons are indicated to the left and soluble Pv-fusion proteins of predicted sizes are marked with an arrow.

Expression of genes encoding five *P. vivax *proteins: PvMsp1-19, PvMsp1-Nter, PvMsp5, PvMsp7, PvDBP-RII, PvCsp-S and GST as control was initially attempted in *E. coli *and wheat germ cell-free expression systems using commercially available vectors (Roche). Yields, however, were very low and highly degraded as detected by Western blot analysis. It was thus decided to incorporate a GST tag into these vectors (Figure 1) as GST increased the solubility and yields of different recombinant proteins [26]. Noticeably, when cloned into these vectors, both expression systems produced readily detectable proteins by Western blot analysis under reducing condition (Figure 4). Proteins expressed by the cell-free *E. coli *system, however, were mostly degraded and showed low amounts of intact proteins with predicted sizes (Figure 4A). In contrast, soluble proteins expressed in wheat germ cell-free system were of predicted sizes and had much less degradation products (Figure 4B). All proteins produced by wheat germ system were affinity-purified to 60-85% and yielded 1-10ug/50ul (Additional file 2). Soluble purified proteins were coupled to individual bioplex beads and coupling efficiency was verified prior to multiplexing using an anti-GST or anti-his antibody (Additional file 3).

### Proteins produced by wheat germ system are recognized by significantly higher number of immune sera than those produced by *E. coli*

**Figure 5:**
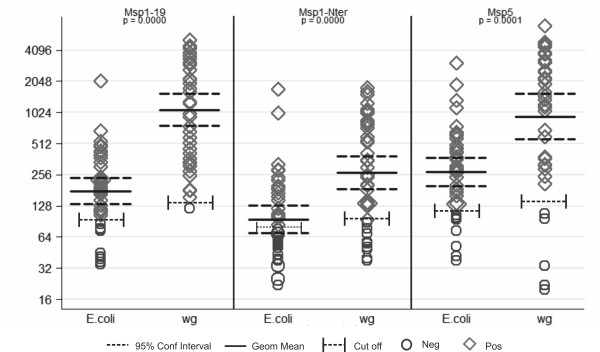
**Comparative analysis of immune responses to *P. vivax *proteins expressed in the *E. coli *and wheat germ cell-free systems by Bioplex**. One μg of each affinity-purified protein was individually coupled to beads and analysed by multiplex assays using immune sera (1:100 dilution) pertaining to 40 different *P. vivax *patients. Fluorescence was determined as the mean fluorescence intensity (MFI). GST values were subtracted from MFI values obtained against individual recombinant proteins and the cut-off defined as the mean value of control sera + 3 standard deviations. Circles represent samples which MFI values were below the cut-off and were considered negative whereas squares represent samples which MFI values were above the cut-off and were considered positive. Geometric means and 95% confidence intervals are shown.

Only three soluble proteins produced in the 50 μl scale in *E. coli *could be purified in a single-step and coupled to Bioplex beads using exactly the same methodology as those produced and purified by wheat germ system. A comparison of naturally acquired humoral IgG responses against these proteins was thus made using immune sera of 40 malaria patients from Brazil known to have large reactivity against PvMSP1 [22]. GST values were subtracted from MFI values obtained against individual recombinant proteins and the cut-off defined as the mean value of control sera +3 standard deviations. Noticeably, proteins produced in the wheat germ system were recognized in significantly higher numbers than those produced in the *E. coli *system (MSP1-19 wheat germ 37/40 (92.5%) vs MSP1-19 *E. coli *19/40 (47.5%), p = 0.000; MSP1-N wheat germ 26/40 (65%) vs MSP1-N *E. coli *8/40 (20%), p = 0.000; MSP5 wheat germ 34/40 (85%) vs MSP5 *E. coli *23/40 (57.5%) (p = 0.001) (Figure 5). Moreover, values of geometric means of all proteins produced in wheat germ system were significantly higher than those produced in *E. coli *system and 95% confidence intervals reinforced such differences. This data demonstrates that identical soluble proteins expressed in wheat germ system and coupled to bioplex beads are better recognized by the same immune sera than those expressed by *E. coli *system.

### Multiplex assays with proteins produced in wheat germ system as an alternative platform for antigen discovery

**Figure 6:**
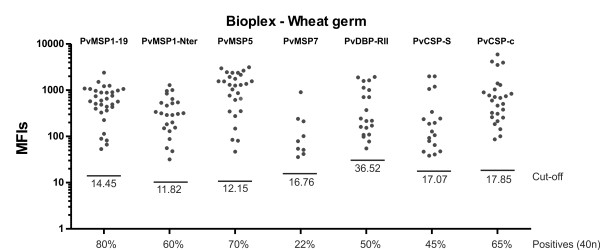
**Naturally acquired humoral IgG immune responses to proteins expressed in the wheat germ cell-free system**. Human IgG antibodies against *P. vivax *recombinant proteins were detected by Bioplex. One μg of wheat germ cell-free-produced proteins were individually coupled to beads and incubated with 40 different individual plasma samples (1:100 dilution) followed by biotinylated human IgG isotypes and detected using PE-streptavidin. Fluorescence was determined as the mean fluorescence intensity (MFI). GST values were subtracted from MFI values obtained against individual recombinant proteins and the cut-off defined as the mean value of control sera + 3 standard deviations. Only positive values above the cut-off are represented as dots.

**Figure 7:**
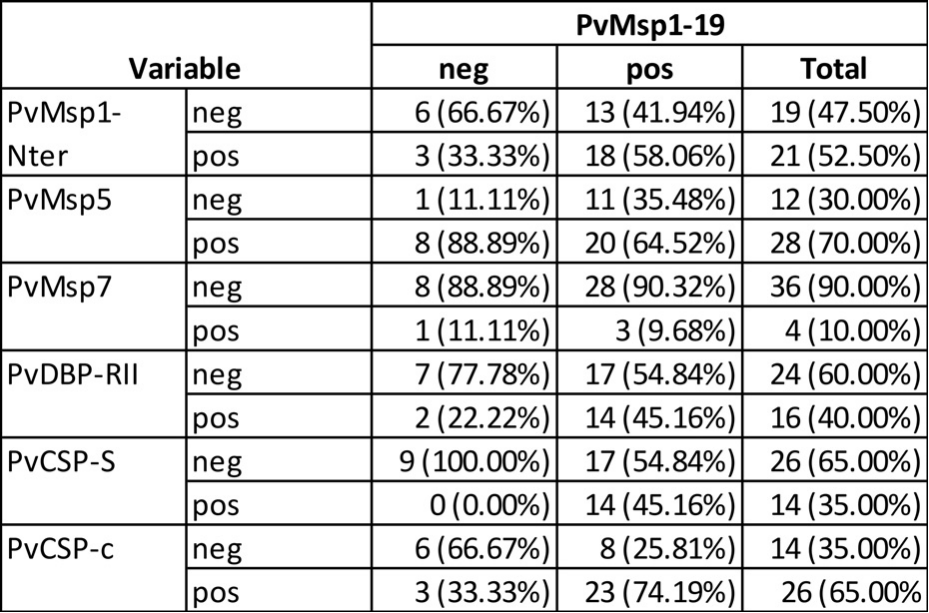
**Cross comparisons of immune responses to PvMSP1-19**. Immune responses to different proteins in the same serum using a 2 × 2 Table on the response distribution over proteins pairs.

To illustrate the use of soluble proteins produced by wheat germ system in a 50 μl scale and multiplexing assays for immuno-epidemiological studies, the responses of other proteins also considered important targets for *P. vivax *vaccine development were determined. These include (besides MSP1-19, MSP1-N, and MSP5), MSP7 [27], PvDBP-RII [28], and CSP [29]. Moreover, a chimeric CSP protein produced in large-scale in wheat germ and containing the two major allele repeats of PvCSP was also included (Figure 3 and Additional file 1). Of note, for this analysis a different group of 40 sera pertaining to other individuals with no particular strong reactivity against PvMSP1 was used [22]. All proteins were first analysed individually using 1 μl of serum diluted 1:100 and then simultaneously using the same quantity and the same dilution. At this dilution, the same sera reacting against PvCSP-S reacted against PvCSP-c even though a subtraction effect was detected in singleplex vs multiplex (Additional file 4). Thus, dilutions of sera in these assays must be taken into consideration to avoid missing immune responders to different alleles of the same protein. The data corroborated the immunogenicity of all these proteins albeit, as expected, to different levels (MSP1-19 80%, MSP1-Nter 60%, MSP5 70%, MSP7 22.5%, PvDBP-RII 50%, PvCSP-S 45% and PvCSP-c 65%) (Figure 6). Moreover, a cross-comparison between responses to the different proteins revealed, for instance, that sera that reacted against PvMSP1-19 also reacted against PvMSP1-N (58.06%), PvMSP5 (64.52%), PvMSP7 (9.68%), PvDBP-RII (45.16%), PvCSP-S (45.16%) and PvCSC-c (74%) (Figure 7). Values for all other cross-comparisons showed similar results with varying percentages of recognition by immune sera against any one particular protein and comparisons with the others (Additional file 5). Of note, there were a significant larger percentage of immune sera reacting against the chimeric CSP (PvCSP-c) as opposed to the one expressing only one allele (PvCSP-S). Moreover, cross-comparison of responses against PvCSP-S and PvCSP-c demonstrated that 92.86% of sera reacted against these two proteins.

## Discussion

Protein arrays containing hundreds to thousands of malarial proteins have been recently reported for antigen discovery [13,15,16]. In these experiments, in vitro transcribed/translated products are directly spotted into solid matrices for analysis and reactivity against human sera. The goal here was developing an alternative simple small-scale method for soluble expression and single-step affinity purification of proteins to be analysed by suspension array technology. To this end, vectors expressing GST fused to the protein of interest were constructed to facilitate soluble expression of *P. vivax *proteins in a 50 μl scale in the cell-free systems of *E. coli *and wheat germ. Soluble proteins were affinity-purified in a single-step, coupled to luminex beads and analysed against immune sera from *P. vivax *patients. Significantly higher number of immune sera reacted against proteins expressed in wheat germ system and multiplexing of five leading vaccine candidates illustrated the use of this method for immuno-epidemiological studies in *P. vivax*.

A major bottle-neck in antigen discovery for vaccine development in malaria is the little success achieved in producing soluble proteins in different cell-based or viral systems. Thus, cell-based *E. coli *and baculo-virus systems have reported expression of soluble malaria proteins anywhere from 6.3-30% [10,30]. In these reports, modifications involving codon optimization, construction of synthetic genes, extensive manipulations of culture conditions, different temperatures, and large culture volumes were needed to achieve solubilisation of proteins [10]. While these methods and expression systems remain highly valuable tools for structural and functional studies, they are difficult to implement on large-scale analysis of malarial proteins for antigen discovery. Noticeably, the development of cell-free expression systems offered a valid and efficient alternative to this objective. In fact, using malarial proteins expressed in cell-free extracts of either *E. coli *or wheat germ and analysed on flat solid arrays with immune sera, recent reports have paved the way for genome-wide antigen discovery of the two major human malaria parasites [13,16]. In these systems, proteins are directly spotted on linear flat surfaces with no formal demonstration of solubility or purity of expressed products. As the goal of these studies is the screening of thousand of antigens in combination with powerful statistical analyses, the presence of false-positives have been considered negligible. Increasing evidence, however, indicates that proteins expressed in wheat germ cell-free system are more suitable for these analyses as they are mostly soluble and retained enzymatic activity [15,31]. Moreover, suspension arrays offer major advantages when compared to protein arrays including nearly solution phase kinetics and total assay sensitivity [21].

The methodology reported here largely facilitates the production of soluble proteins in a small-scale compatible with automation and in quantities allowing analysis of hundreds of sera (roughly 1 μg of soluble/affinity-puried protein can be used to screen approximately 250 sera) using suspension arrays. To illustrate this, we expressed five leading vaccine candidates against two different life stages, the pre-erythrocytic stages (CSP) and asexual blood stages (MSPs and DBP). CSP is considered a leading vaccine candidate in *P. falciparum *[32] and the homologous protein has entered clinical trials in *P. vivax *[8]. PvCSP contains two major allele forms, PvCSP-VK210 [29] and PvCSP-VK247 [23]. We expressed PvCSP-VK210 in the 50 μl scale and also tested a chimerical protein composed of both major alleles (PvCSP-c) produced in large-scale. Both proteins were readily recognized by immune sera even though significantly larger number of sera reacted against the PvCSP-c protein representing these two major alleles. The fact that lower number of sera reacted against PvCSP-S could be due to lower amounts of full CSP coupled to the beads as there was a major degradation product detected by SDS-PAGE (Additional file 2). Alternatively, these results are due to the presence of both major alleles in this chimerical protein as both readily circulate in the Brazilian Amazon [33]. In the absence of further evidence, this remains to be investigated.

Proteins expressed during the asexual blood stages are responsible for pathology associated with malaria and are, therefore, the target of intense efforts to discover antigens for vaccination. Naturally acquired humoral immune responses against merozoite surface proteins were thus initially analysed as they are involved in invasion to red blood cells and are considered candidates to develop sub-unit vaccines against malaria [27]. In particular, MSP1, MSP5 and MSP7 were studied as different reports from these proteins indicate their potential in vaccine development [8]. MSP1 and MSP5 are encoded by single gene whereas MSP7 pertains to a highly variant multi-allelic family [9]. As expected, results demonstrated that MSP proteins are immunogenic in natural infections. Moreover, results confirmed that MSP1-19 is more immunogenic than MSP1-N [20] and that in spite of MSP5 being highly polymorphic [34], it is also highly immunogenic. Furthermore, in line with being a multigene family differentially expressed during blood stages [35], reactivity against MSP7 was lower than MSP1 or MSP5. In addition to MSPs, the response against the Duffy binding protein region II (PvDBP-II) a leading vaccine candidate against *P. vivax*, was also analysed. PvDBP-II is cysteine-rich and requires a complex series of steps to fold it correctly [28]. Results confirmed the immunogenicity of PvDBP-II in natural infections as previously reported using sera from adult patients in Brazil [33]. Whether these antibody responses against different asexual blood stages are inhibitory as shown for the PvDBP-II [36] awaits the development of functional assays.

In summary, expression of soluble proteins from *P. vivax *for analysis in multiplexing assays using the wheat germ cell-free system in a 50 μl scale has been achieved. In addition to the five leading vaccine candidates illustrating here this methodology, several other proteins including subtelomeric variant Vir and PfamD proteins, Pvs48/45, and several hypothetical antigenic proteins, have been solubly expressed at this scale. Up to 100 proteins can be presently coupled to different beads and analysed simultaneously with as little as one microliter of immune sera. Prospective longitudinal studies from endemic regions with different degrees of transmission and clinical immunity using this methodology will complement studies using protein arrays and will accelerate antigen discovery and vaccine development in *P. vivax*.

## List of abbreviations

GST: Glutathione S-Transferase; MFI: Median fluorescent intensity; PE: phycoerythrin

## Competing interests

The authors declare that they have no competing interests.

## Authors' contributions

ER contributed to write the manuscript, to design and to conduct the experiments. ST and TT made substantial constructive advice in the initial design of the project and constructed as well as expressed the PvCSP chimerical protein. SS performed statistical analyses. MVGL made advice in the last design of the project and critically read the manuscript. CFB and HAP conceived this study and contributed to write the manuscript and to design experiments. All authors read and approved the final manuscript.

## Supplementary Material

Additional file 1 **Proteins and primers used in this study**. ID, identification. AA, amino acids. MW, molecular weight. IP, isoelectric point. Columns to the right represente GST-fusion proteins. Sequence of primers. Click here for file

Additional file 2 **Purification of proteins from wheat germ lysates**. Soluble fractions from wheat germ extracts were applied to a Glutathione Sepharose^® ^4B column equilibrated with PBS. Columns were washed with PBS and bound GST-fusion proteins eluted with 10 mM glutathione in 50 mM Tris-HCl, pH 8.0. Collected fractions were analysed by SDS-PAGE. Molecular weights of standard control proteins are indicated and soluble GST-fusion proteins are marked with an arrow. Click here for file

Additional file 3 **Coupling efficiency of proteins to activated beads**. Specific detection of 8 tagged Pv-protein on beads. Protein were expressed in wheat germ cell free system, purified and 1 ug bound to the beads. Prior to multiplexing, protein coupling was verified by incubating coupled beads with mouse anti-Gst or anti-his (for PvCSP-c) antibody followed by biotinylated anti-mouse IgG. The biotinylated antibodies were detected using PE-streptavidin with the Luminex analyzer beads, and fluorescence was determined in the mean fluorescence intensity (MFI). Click here for file

Additional file 4 **Comparative analysis of immune responses to PvCSP-S and PvCSP-c by singleplex and multiplex**. Immune sera were analysed in a single-vs multiplex assay. Values above 1 indicates increased response as multiplex assay. Values below 1 indicates that there was a decrease of the response as multiplex assay. Click here for file

Additional file 5 **Comparative analysis between responses to different proteins in the same serum using 2 × 2 tables**. Click here for file
