# Supplementary figures and images for: Plasmodium vivax: comparison of immunogenicity among proteins expressed in the cell-free systems of Escherichia coli and wheat germ by suspension array assays
Source: Malar J. 2011 Jul 14;10:192. doi: 10.1186/1475-2875-10-192 (PMC3224337; doi:10.1186/1475-2875-10-192)

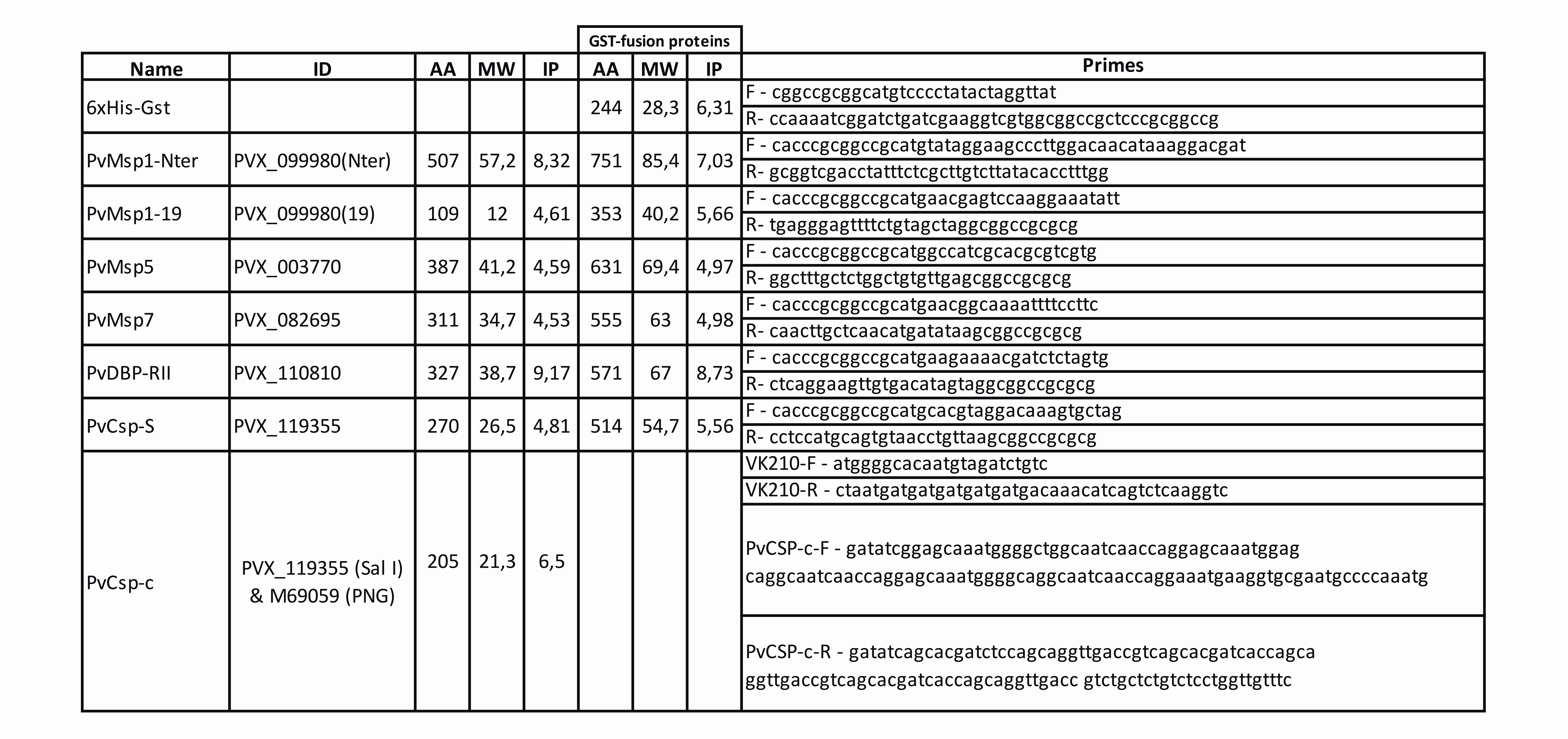

Supplement: Additional file 1 — Proteins and primers used in this study. ID, identification. AA, amino acids. MW, molecular weight. IP, isoelectric point. Columns to the right represente GST-fusion proteins. Sequence of primers. [file 1475-2875-10-192-S1.TIFF]

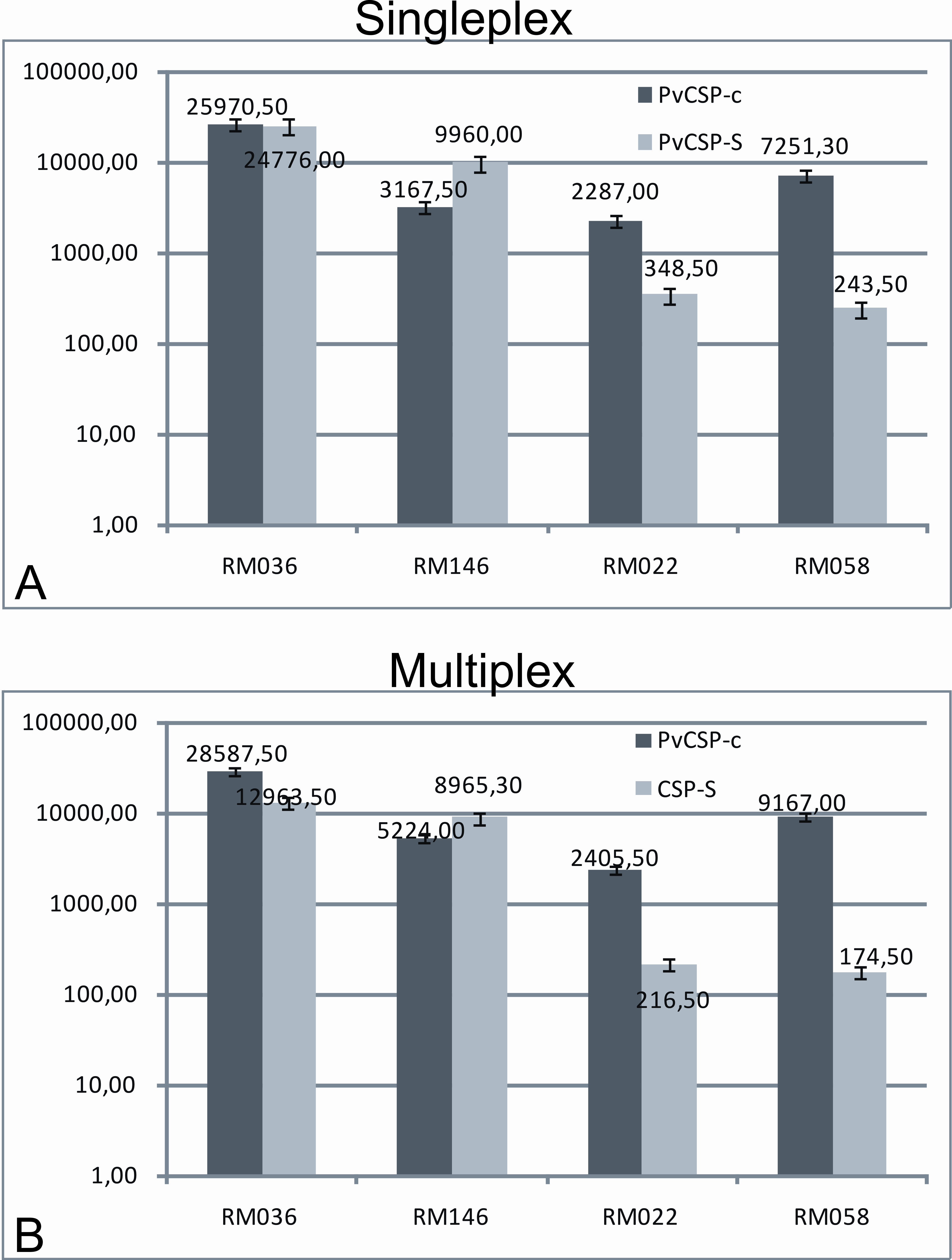

Supplement: Additional file 4 — Comparative analysis of immune responses to PvCSP-S and PvCSP-c by singleplex and multiplex. Immune sera were analysed in a single-vs multiplex assay. Values above 1 indicates increased response as multiplex assay. Values below 1 indicates that there was a decrease of the response as multiplex assay. [file 1475-2875-10-192-S4.TIFF]
